# The Mechanism of Fraxetin as a Sustainable Fungicide for Larch Shoot Blight: Lipid Peroxidation and Oxidative Stress in *Neofusicoccum laricinum*

**DOI:** 10.3390/jof11100724

**Published:** 2025-10-08

**Authors:** Shuang Zhang, Ruizhi Zhang, Rui Xia, Xinyan Chen, Jiarui Chen, Yuchun Yang, Majid Mujtaba, Danlei Li, Feng Wang

**Affiliations:** 1 Key Laboratory of Alien Forest Pest Detection and Control-Heilongjiang Province, College of Forestry, Northeast Forestry University, Harbin 150040, China; zhangshuang1666@163.com (S.Z.); zhangruizhi@nefu.edu.cn (R.Z.); xiarui@nefu.edu.cn (R.X.); cxy0201@nefu.edu.cn (X.C.); chenjr220924@163.com (J.C.); young87654321@163.com (Y.Y.); majid.mujtaba.057@gmail.com (M.M.); 2 Key Laboratory of Sustainable Forest Ecosystem Management-Ministry of Education, College of Forestry, Northeast Forestry University, Harbin 150040, China; 3 State Key Laboratory of Tree Genetics and Breeding, College of Forestry, Northeast Forestry University, Harbin 150040, China

**Keywords:** fraxetin, larch shoot blight, *Neofusicoccum laricinum*, antifungal mechanism

## Abstract

Larch shoot blight, caused by *Neofusicoccum laricinum*, threatens global larch resources, while conventional chemical control is constrained by pollution and resistance. To address this gap, we integrated metabolomics, transcriptomics, and antifungal efficacy assays to identify Fraxetin, a disease-induced phytoalexin, and to elucidate its antifungal activity and mechanism. Metabolomics showed infection-triggered accumulation of Fraxetin in resistant *Larix olgensis* shoots. Antifungal experiments showed that within the range of 68–1088 μg/mL, the optimal antifungal concentration was 1088 μg/mL. When inoculated larches were treated with 1088 μg/mL Fraxetin, the maximum inhibition rate of pathogen growth reached 66.67% within 12 days, and the symptoms of the treated plants were alleviated. Transcriptomics revealed activation of damage responses, disruption of oxidative homeostasis, and compromised membrane integrity in the pathogen under Fraxetin treatment. Physiological measurements confirmed increased lipid peroxidation, redox collapse, membrane leakage, and reduced fungal viability. These findings indicate a lipid peroxidation–mediated oxidative–membrane mode of action and support the potential of plant-derived Fraxetin for more sustainable management of larch shoot blight.

## 1. Introduction

Larch shoot blight, caused by *Neofusicoccum laricinum*, is a significant fungal disease that threatens the growth and wood quality of larch forests worldwide, resulting in considerable ecological and economic losses [1]. The disease primarily affects the young shoots and branches of larch, leading to stunted larch growth, wood damage, and a decrease in forest ecosystem stability [2]. Currently, chemical control is the main strategy for managing larch shoot blight, complemented by forest management practices such as proper stand management, improved ventilation and light penetration, and timely removal of infected shoots. Although chemical fungicides have been effective in controlling the disease over the long term, their continued use has resulted in environmental pollution and the development of pathogen resistance. The widespread application of traditional chemical fungicides has been accompanied by growing concerns regarding resistance, environmental pollution, and fungicide residues, rendering existing control methods inadequate for sustainable forestry development [3]. As a result, the search for environmentally friendly and efficient alternative control methods has become an urgent priority in plant protection.

Plants have evolved a complex defense system to resist pathogen invasion and environmental stress, which includes pathogen recognition, signal transduction, and the activation of defense responses [4,5]. This system is effective in responding to a variety of pathogens and plays a critical role in the relationships between plants and pathogens [6]. Notably, this multi-layered defense mechanism not only involves the rapid activation of signaling pathways but also relies on the spatiotemporal accumulation of specific metabolites to ensure precise defense [7]. Among these metabolites, secondary metabolites form the material basis for plant chemical defense. Due to their diverse chemical structures and biological activities, they are essential in protecting plants from pathogenic invasion [8]. Phytoalexins, key secondary metabolites produced during the plant’s long-term adaptation to environmental stress, play a central role in this defense system [9]. These metabolites, characterized by strong lipophilicity and low molecular weight, effectively resist both biotic and abiotic stresses. Phytoalexins perform a dual role in plant disease resistance: they form a direct chemical barrier to inhibit or prevent pathogen infection and act as signaling molecules that regulate plant immunity, activating relevant disease resistance pathways. Thus, they serve as crucial components in the regulation of plant defense responses [10]. These phytoalexins can serve as potential biomarkers for resistant breeding and as candidate compounds for the development of botanical fungicides.

As biomarkers of tree resistance, phytoalexins offer a promising strategy for the continuous protection of forests and crops by enabling the effective screening of resistant plants—derived from the type and content of these compounds. Through their antifungal mechanisms, which have been optimized during evolution, and their species-specific structural diversity (e.g., flavonoids, terpenes, and stilbenes), phytoalexins protect plants from fungal pathogens. This strategy not only accelerates the development and application of botanical fungicides but also improves plant disease resistance through targeted resistance breeding. Existing research has established that various catechol-type phytoalexins are integral to disease resistance mechanisms. Catechol (C_6_H_6_O_2_) is a benzenediol compound characterized by a benzene ring with two hydroxyl groups as substituents [11,12]. It is widely acknowledged that certain phytoalexins, such as catechol [13], umbelliferone [14,15], furanocoumarins, daphnetin, and Fraxetin, function as phytoalexins with significant fungicidal properties in plants [16,17,18].

Fraxetin (7,8-dihydroxy-6-methoxycoumarin) is a hydroxycoumarin containing a 6-methoxy group and catechol, with a molecular weight of 208.17. This compound is found in various plant families, including *Oleaceae* spp., *Sapindaceae* spp., and *Euphorbiaceae* spp., with a notable presence in *Cortex fraxini* [19]. Fraxetin has garnered interest in antimicrobial research due to its bioactive properties. Fraxetin significantly inhibited the growth of *Staphylococcus aureus* by disrupting nucleic acids and proteins and preventing topoisomerase from binding to DNA [20]. Furthermore, Fraxetin demonstrated antifungal activity against *Bipolaris maydis*, *Sclerotium rolfsii*, and *Alternaria solani* [21]. Fraxetin demonstrates significant antifungal properties, suggesting its dual role as a potential biomarkers for tree resistance and as a promising candidate for the development of novel botanical fungicides. Despite its potential, the precise mechanisms underlying Fraxetin’s role in larch defense against larch shoot blight remain incompletely understood. Key knowledge gaps include the antifungal mechanism of Fraxetin and its potential as a candidate for botanical fungicides. Consequently, elucidating the chemical defense mechanisms employed by Fraxetin against larch shoot blight, with a particular emphasis on its antifungal properties, is of significant scientific importance. This study, therefore, aims to elucidate the specific antifungal mechanism of the phytoalexin Fraxetin against *N. laricinum* and to determine the relationship between its accumulation and host resistance.

Building upon the prior identification of a resistant *Larix olgensis* cultivar against larch shoot blight, we designed a screening study focused on phytoalexins and selected Fraxetin as a candidate compound. This work aims to evaluate Fraxetin’s ability to inhibit the mycelial growth of *N. laricinum* and to test the hypothesis that perturbation of fungal hyphal cell-membrane lipids—particularly lipid peroxidation—may mediate its antifungal activity. By defining Fraxetin’s antifungal potential and probing its putative mechanism, the study is intended to inform sustainable forestry practices and provide an environmentally friendly strategy for managing larch shoot blight. We will also assess Fraxetin’s toxicity profile and pathogen-suppression potential to gauge its suitability as a botanical fungicide and its prospects as an alternative to chemical fungicides. Furthermore, we will characterize Fraxetin accumulation patterns in larch tissues to evaluate their utility as biomarkers to support breeding strategies for resistant larch.

## 2. Materials and Methods

### 2.1. Materials

The resistant *L. olgensis* (NL5) and susceptible *L. olgensis* (NL7) were provided by the Key Laboratory of Alien Forest Pest Detection and Control-Heilongjiang Province, Northeast Forestry University [22].

The *N. laricinum* HLJ001 strain, isolated from diseased *L. olgensis* in Shangzhi, Heilongjiang Province, was identified as *N. laricinum* based on morphological and molecular biological methods [23].

Fraxetin (purity ≥ 98%) used in this study was purchased from Shanghai yuanye Bio-Technology Co., Ltd. (Shanghai, China).

### 2.2. Metabolomics Determination and Fraxetin Content Determination

To evaluate the secondary metabolites of both susceptible and resistant *L. olgensis*, untargeted metabolomics using LC-MS/MS was conducted to quantify the metabolites in their shoots before and after inoculation, and targeted metabolomics analysis was performed to determine the content of Fraxetin. The materials and methods were as follows: Instruments used included an Agilent 1100 high-performance liquid chromatograph, Compass C18 (2) reversed-phase chromatographic column (250 mm × 4.6 mm, 5 μm), ultrasonic cleaner, grinder, centrifuge, constant-temperature water bath, and vortex mixer; reagents were Fraxetin standard (Shanghai Yuanye Biotechnology Co., Ltd., Shanghai, China, purity ≥ 98%), HPLC-grade methanol (Osenbach (Tianjin) Technology Development Co., Ltd., Tianjin, China, purity ≥ 99.9%), formic acid, and ultrapure water. For sample pretreatment, samples were collected as required, ground into powder in liquid nitrogen, with approximately 0.2 g of the powdered sample weighed, mixed with 1.0 mL of 80% methanol–water solution, further ground into a slurry using a grinder, ultrasonicated for 1 h, and centrifuged to collect the supernatant, which was then filtered through a needle filter and stored for subsequent analysis. The liquid chromatography conditions were set as follows: Agilent 1100 high-performance liquid chromatograph with a detection wavelength of 340 nm (Agilent Technologies (China) Co., Ltd., Beijing, China), Compass C18 (2) reversed-phase chromatographic column (250 mm × 4.6 mm, 5 μm) (Chromai (Beijing) Technology Co., Ltd., Beijing, China), column temperature of 30 °C, flow rate of 1.0 mL/min, injection volume of 10 μL, and mobile phase of methanol:0.1% formic acid in water = 30:70 (*v*/*v*). For standard curve preparation, a precise amount of Fraxetin standard was weighed and dissolved in methanol to prepare 5–6 standard solutions with different mass concentrations, each of which was analyzed under the above chromatographic conditions to record peak areas, and a standard curve was constructed with peak area as the ordinate and concentration as the abscissa, with the linear range and correlation coefficient calculated.

### 2.3. Evaluation of Fraxetin’s Fungicidal Activity and Its Efficacy in Larch Shoot Blight Control

To evaluate the antifungal activity of Fraxetin against *N. laricinum*, targeted metabolomic analysis first determined the baseline Fraxetin content in resistant larches, with a minimum concentration of 68 μg/mL. Based on this, PDA media with gradient concentrations of Fraxetin (68–1088 μg/mL) were prepared using the doubling method. This setup, spanning a relatively wide range, aimed to systematically investigate Fraxetin’s inhibitory effects on *N. laricinum* and clarify the concentration threshold for its optimal inhibitory activity, thereby providing theoretical and practical references for controlling larch shoot blight. Control plates contained an equal volume of DMSO added to PDA. A 5-mm-diameter mycelial disc was inoculated onto each drug-containing PDA medium with different concentrations, with three replicates set for each concentration treatment [2]. After inoculation, all Petri dishes were incubated in the dark at a constant temperature of 25 °C for 5 days. During this period, the colony diameter was measured every 24 h, and the mycelial growth morphology, coverage range, and color changes were photographed and recorded, with three biological replicates per group. The colony area was calculated based on the measured diameter to determine the inhibition rate (I).

The inhibition rate (I) was calculated using the formula:

(1) $$I=\frac{{\pi \left(\frac{D_{1}-0.5}{2}\right)}^{2}-{\pi \left(\frac{D_{2}-0.5}{2}\right)}^{2}}{{\pi \left(\frac{D_{1}-0.5}{2}\right)}^{2}-{\pi \left(\frac{0.5}{2}\right)}^{2}}\times 100\%$$

where *D*_1_ and *D*_2_ represent the colony diameters of the control (CK) and treatment groups.

Larch shoots were selected, cleaned, then surface-disinfected with 75% ethanol, then rinsed 3 times with sterile water, and dried with sterile filter paper. Then, a 0.5 cm × 0.5 cm fungal cake was inoculated after creating a uniform wound (0.5 cm × 0.5 cm) on each shoot with a sterile blade. The experiment was divided into two groups: for the experimental group, 1.2 days post-inoculation (dpi), shoots were sprayed with 1 mL of Fraxetin treatment (concentration: 1088 μg/mL, solvent: DMSO) on the wound surface and on the epidermis of larch shoots within 1 cm radius around the wound, and 1 mL of Fraxetin treatment was sprayed on the wound surface; the CK group was sprayed with the same volume of DMSO treatment, diluted with sterile water, and all other operations were the same as the experimental group, with three biological replicates per group. The two groups of shoots were incubated under the same greenhouse conditions, their growth was recorded regularly with a camera, and the disease severity was assessed at 8 dpi and 14 dpi after inoculation, while the occurrence, spread and disease severity of the lesions were observed.

Infected shoots were classified into the following grades:

Grade 0: healthy;

Grade I: stem de-greened, few needles shed;

Grade II: stem yellow-brown, approximately 50% needle loss, shoot tip slightly drooping;

Grade III: stem brown, most needles shed, shoot tip drooping;

Grade IV: stem dark brown, all needles shed except for a cluster of purple-gray necrotic needles at the tip.

The disease index (DI) was calculated.

(2) $$DI=\left[\frac{\left(0n_{0}+1n_{I}+2n_{II}+3n_{III}+4n_{IV}\right)}{4n}\right]\times 100\;$$

In these formulas, the number of plants at each disease grade (*n*_0_–*n_IV_*) and the total number of plants surveyed (n) were used to calculate DI.

### 2.4. Physiological Analysis of Fraxetin’s Antifungal Mechanism

Cell membrane integrity was evaluated by Propidium Iodide (PI) staining to detect the red-stained ratio of *N. laricinum* mycelia after treatment with 68–1088 μg/mL Fraxetin, observed under a fluorescence microscope [24].

To analyze the level of lipid peroxidation, the dynamic changes of Malondialdehyde (MDA) content were determined after Fraxetin treatment [25]. The experiment was performed strictly in accordance with the instructions of the MDA Assay Kit and the absorbance was measured at wavelengths of 532 nm and 600 nm.

The activity of the antioxidant enzyme Catalase (CAT) was assayed [26,27]. The experimental procedures were carried out with reference to the instructions of the CAT Assay Kit and the detection was conducted at a wavelength of 510 nm.

The activity of superoxide dismutase (SOD) was determined [28,29]. The experimental operations were performed in accordance with the instructions of the SOD Assay Kit.

### 2.5. Multi-Omics Mechanism Investigation of Fraxetin’s Antifungal Action

Transcriptomics: Total RNA was extracted from *N. laricinum* mycelia treated with 1088 μg/mL Fraxetin for 12 h and 24 h using the TRIzol method. RNA sequencing was performed on the Illumina NovaSeq platform. Differential gene expression was identified based on the criteria (|log_2_FC| ≥ 1, *p* < 0.05), followed by KEGG enrichment analysis [2]. All transcriptome data were analyzed using the ggplot2 package in the R language (version 4.1.3).

### 2.6. Data Analysis

Time-dependency models were analyzed using linear or nonlinear fitting to assess the accumulation dynamics (BMTL/BMTU calculations). Statistical analyses included Fisher’s exact test and PLS regression (R^2^/Q^2^ to assess the correlation between gene expression and metabolite accumulation). Dose–response analyses were performed to quantify the inhibition rates and determine the dose–effect curve (AUC values). In field trials, disease incidence and disease index were statistically evaluated in the susceptible *L. olgensis* treated with Fraxetin.

## 3. Results

### 3.1. Fraxetin Functions as a Key Phytoalexin Mediating Disease Resistance in Larch

**Figure 1:**
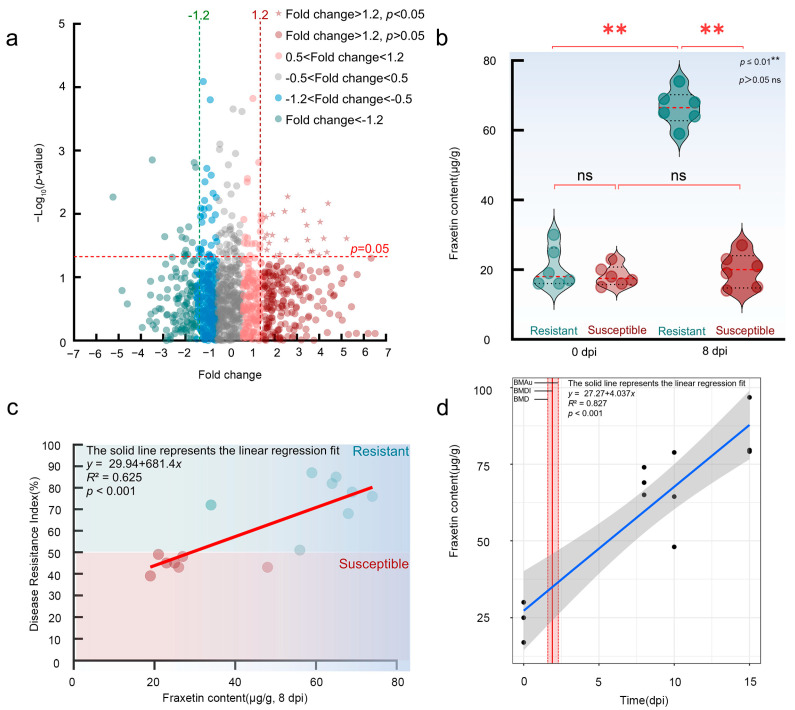
Fraxetin Synthesis, Induction, and Spatiotemporal-specific Accumulation in *L. olgensis*. (**a**) Metabolomic profiling identifies significantly upregulated metabolites in resistant *L. olgensis* (fold change ≥ 1.20, *p* < 0.05), which are enriched in phytoalexin-related pathways. Phenylpropanoid biosynthesis pathway in *L. olgensis*, highlighting the coumarin. (**b**) Induced Fraxetin synthesis in resistant *L. olgensis* following *N. laricinum* inoculation. (**c**) Correlation of Fraxetin content with disease resistance index in *L. olgensis.* (**d**) Spatiotemporal accumulation of Fraxetin in resistant larch in response to pathogen exposure.

Untargeted and targeted metabolomics analyses identified Fraxetin as a predominant phytoalexin associated with resistance *L. olgensis* (Fraxetin’s 2D structure is shown in Appendix A). Differential metabolomic profiling revealed 24 secondary metabolites exhibiting significant alterations following inoculation (fold change ≥ 1.20, *p* < 0.05; Figure 1a), suggesting that resistance is mediated by the synergistic action of multiple compounds. Among these metabolites, Fraxetin—a hydroxycoumarin derivative originating from phenylpropanoid biosynthesis—demonstrated the strongest correlation with resistance phenotypes (*R*^2^ = 0.62).

The synthetic characteristics of phytoalexins were confirmed through the analysis of three aspects: their inducibility, their correlation with disease resistance, and their spatiotemporal-specific accumulation.

Firstly, it was found that inoculation with *N. laricinum* induced the biosynthesis of Fraxetin in resistant *L. olgensis*. Before inoculation, the basal Fraxetin content did not differ significantly between resistant (20.5 ± 5.75 μg/g) and susceptible larches (18.17 ± 2.93 μg/g) (Student’s *t*-test, *p* > 0.05). After inoculation with *N. laricinum*, the Fraxetin content in resistant larches increased sharply, reaching 66.5 ± 5.09 μg/g at 8 dpi, which was approximately 3 times the original level (Student’s *t*-test compared to its own pre-inoculation level, *p* < 0.001). In contrast, susceptible larches showed no significant change in Fraxetin content after inoculation (19.83 ± 4.92 μg/g; Student’s *t*-test compared to its own pre-inoculation level, *p* > 0.05), indicating a pathogen-specific inducible defense response in resistant varieties (Figure 1b).

Secondly, a correlation analysis between Fraxetin content and the disease resistance index confirmed a significant positive correlation. Targeted metabolomic analysis of 15 *L. olgensis* larches revealed a Pearson correlation coefficient of *r* = 0.72 (*p* < 0.001) between Fraxetin content (at 8 dpi) and the disease resistance index. A linear regression model indicated that Fraxetin content explained 62.5% of the variation in disease resistance phenotypes (*R*^2^ = 0.62, *p* < 0.004) (Figure 1c). Further analysis showed that resistant larches with Fraxetin content ≥ 60 μg/g exhibited an average disease resistance index of 77.80 ± 7.30, significantly higher than that of the low-accumulation group (resistance index: 53.60 ± 14.60; *t*-test, *p* < 0.006). Normality tests confirmed that both Fraxetin content (Shapiro–Wilk, *p* = 0.15) and the disease resistance index (Shapiro–Wilk, *p* = 0.09) adhered to a normal distribution assumption (*p* > 0.05).

Lastly, the spatiotemporal specificity of Fraxetin accumulation was confirmed. Statistical analysis revealed that during pathogen exposure, Fraxetin concentration increased linearly with prolonged exposure time, exhibiting a significant time-dependent response (linear model: *p* = 0.21, BMD = 1.20, BMDI = 1.60, BMDu = 1.90; Figure 1d). BMD modeling estimated that the duration necessary to achieve a biologically significant accumulation of Fraxetin was 1.20 dpi. The corresponding 90% confidence interval (BMDI) for this estimate ranged from 1.60 to 1.90 dpi, demonstrating the precision of the calculation. These findings confirm that Fraxetin accumulation is a specific and temporally regulated response to infection. The lack-of-fit test result (*p* = 0.21) indicated that the data supported a linear model for Fraxetin accumulation, biologically reflecting the stability of its response mechanism. Fraxetin was primarily enriched at pathogen infection sites, with detection levels in distal tissues accounting for less than 5% of those in hotspot regions. The Lack-of-Fit Test indicated that the linear model between Fraxetin concentration and exposure time was not significantly rejected by the data (*p* > 0.05), supporting a linear accumulation pattern. This suggests that the synthesis or metabolic pathways of Fraxetin exhibit high stability during pathogen exposure without nonlinear fluctuations (e.g., plateau or acceleration phases), implying that its response mechanism may rely on a persistently activated single regulatory pathway. Further analysis showed that Fraxetin accumulation was dose-dependent on pathogen exposure, with a clear effect threshold, suggesting that it may act as a key signaling molecule in plant defense, mediating dose-sensitive disease resistance responses. As pathogen exposure time increased, Fraxetin concentration continued to rise, indicating that its synthetic pathways (such as chalcone synthase and prenyltransferase in the phenylpropanoid pathway) were not subject to negative feedback regulation. This continuous accumulation trend further supported the linear relationship between Fraxetin accumulation and exposure time (*p* = 0.23) and the dose effect (BMD = 1.20). These results indicated that Fraxetin accumulation mechanisms were highly stable and predictable, reflecting the strategy of plants to achieve precise defense through directional metabolic flow regulation under pathogen stress.

### 3.2. Dose- and Time-Dependent Antifungal Activity of Fraxetin Against N. laricinum

**Figure 2:**
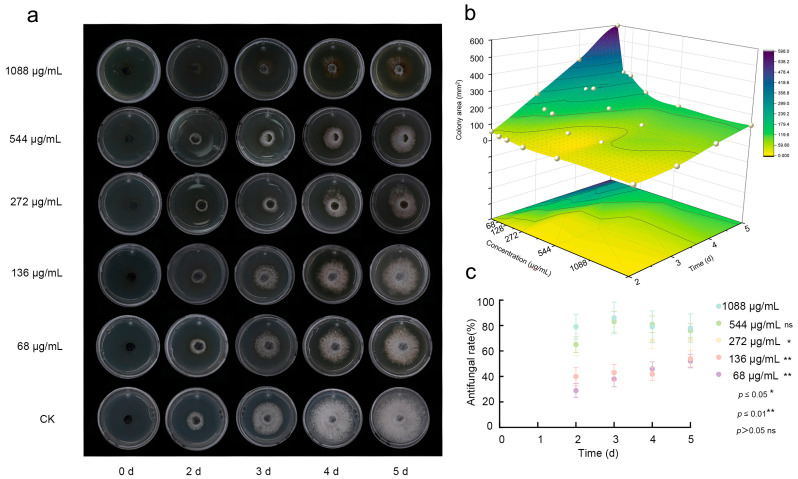
Concentration-Dependent Antifungal Activity of Fraxetin Against *N. laricinum.* (**a**) Inhibition of *N. laricinum* colony growth over 1–5 d under Fraxetin treatment (68–1088 μg/mL). Control: Untreated *N. laricinum*. (**b**) 3D response surface plot of Fraxetin’s antifungal activity, illustrating the effects of dose and time on the colony area of *N. laricinum*. (**c**) Scatter plot of the antifungal activity of Fraxetin.

Fraxetin exhibited concentration-dependent antifungal activity against *N. laricinum*. Dose–response assays, conducted over a range of 68–1088 μg/mL, demonstrated a progressive inhibition of fungal growth correlating with both increased concentration and extended incubation time. At a concentration of 1088 μg/mL, complete suppression of mycelial growth was observed within 5 dpi (Figure 2a,b). This indicates significant antifungal activity was demonstrated, with an inhibition rate of 77.68% achieved by the 5 dpi (Figure 2c). Although the medium- and low-concentration groups did not achieve the same level of activity as the high-concentration group, they still exhibited noticeable inhibitory effects over time. In contrast, the medium-concentration group (272 μg/mL and 544 μg/mL) displayed some variability in antifungal rates over time, with an overall inhibition effect that was less pronounced than that of the high-concentration group. The low-concentration groups (136 μg/mL and 68 μg/mL) demonstrated even lower inhibition rates; however, these rates gradually increased over time. In conclusion, the findings of this study demonstrate that Fraxetin effectively inhibits the growth of *N. laricinum*, exhibiting dose- and time-dependent antifungal properties.

### 3.3. Efficacy of Exogenous Fraxetin in Controlling Larch Shoot Blight

In the previous section, we determined that within the concentration range of 68–1088 μg/mL, the concentration of Fraxetin exhibiting the best inhibitory efficacy against *N. laricinum* is 1088 μg/mL. Therefore, we sprayed 1088 μg/mL Fraxetin on inoculated larches to investigate its inhibitory efficacy on larch shoot blight.

**Figure 3:**
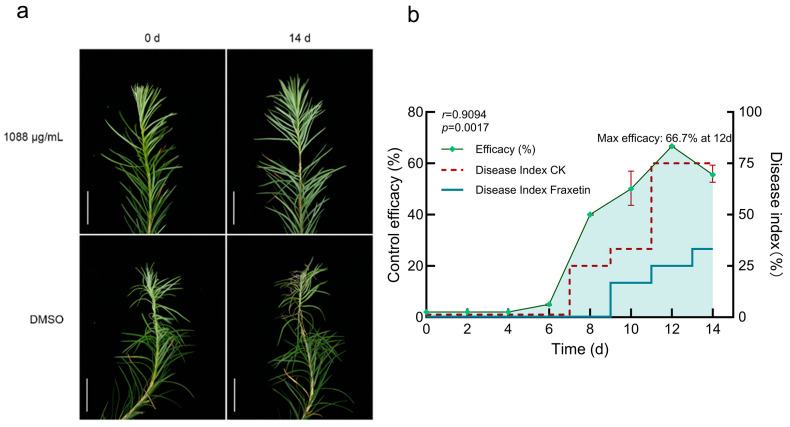
Exogenous Fraxetin Suppresses Lesion Development in *N. laricinum*-Infected Larch Shoots. (**a**) Disease symptoms in larch shoots inoculated with *N. laricinum* and treated with 1088 μg/mL Fraxetin or DMSO at 0 and 14 d. Scale bars: 2.50 cm. (**b**) Time-dependent efficacy of Fraxetin treatment after DMSO correction at 14 d. Mann–Whitney U test at 14 d showed significantly lower disease severity in Fraxetin-treated larches compared to controls (U = 0, *p* < 0.05), indicating significant therapeutic efficacy of Fraxetin.

Exogenous fraxetin (1088 μg/mL in DMSO) significantly mitigated the progression of larch shoot blight. DI was assessed at multiple time points for both the Fraxetin-treated and the DMSO solvent CK groups. Symptom progression was observed as follows: Within the first 7 dpi, no significant differences were observed between the groups. Current-year shoots in both groups remained green without notable needle abscission, with no macroscopic signs of disease development. At 8 d, divergence became evident: CK plants showed initial blight symptoms, including shoot chlorosis and slight needle abscission (grade I), whereas Fraxetin-treated plants remained asymptomatic (grade 0). Accordingly, DI increased in CK but remained low in the treated group. By 10 d, disease severity in the CK group had advanced to grade II, characterized by a yellow-brown shoot discoloration and continued needle abscission. In contrast, the treated group exhibited only mild symptoms (grade I), such as shoot chlorosis and minor needle drop. The DI was significantly higher in the control group than in the treated group. By 14 d, CK plants were severely blighted (grade III), with shoots turning dark brown, shoot tips drooping due to desiccation, and extensive needle loss, resulting in a DI of 75.00% (Figure 3a). Conversely, treated plants remained stable, showing only slight needle abscission around inoculation sites. The retained needles were light green without conspicuous browning, the cortex remained smooth without wrinkling, and lesions did not expand, corresponding to grade I–II and a DI of 33.25%. Upon excluding the influence of DMSO, the treatment efficacy exhibited a time-dependent pattern, with values increasing from 50.00% on 10 d to 66.67% on 12 d and then slightly decreasing to 55.56% on 14 d (Figure 3b). The Mann–Whitney *U* test was employed to evaluate the differences in disease indices between the Fraxetin-treated and CK groups at 14 d. Following the ranking of the combined data, the rank sums were computed as follows: Fraxetin-treated group rank sum = 6, CK group rank sum = 15. The U statistic was calculated to be U = 0, and the significance test (U = 0 ≤ critical value U = 0, *p* < 0.05) confirmed a statistically significant efficacy of Fraxetin treatment. These results underscore Fraxetin’s potential as a promising phytoalexin, with significant therapeutic efficacy against larch shoot blight. Consequently, Fraxetin emerges as a viable candidate for development as a botanical fungicide.

### 3.4. Transcriptomic Insights into Membrane Damage and Oxidative Stress Modulation in N. laricinum by Fraxetin

Transcriptomic analysis revealed that Fraxetin disrupts *N. laricinum* through three synergistic mechanisms: the induction of a damage response, the dysregulation of oxidative stress, and the compromise of membrane integrity. Collectively, Fraxetin perturbs the fungal defense network by targeting multi-pathways, disrupting oxidative defense, energy supply, and membrane stability, culminating in cellular dysfunction and ultimately resulting in cell death.

**Figure 4:**
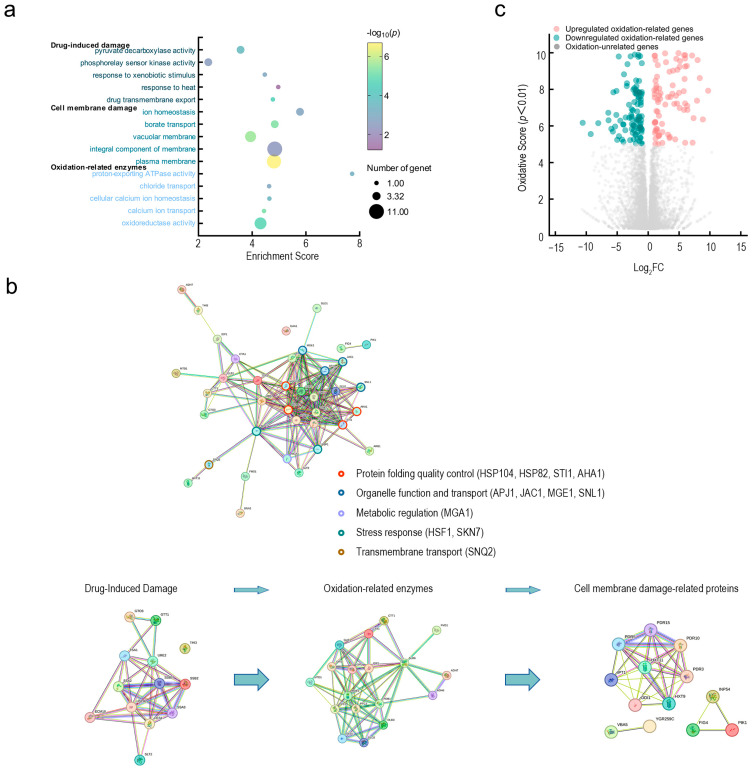
Transcriptomic mechanisms of Fraxetin’s antifungal action against *N. laricinum*. (**a**) GO enrichment analysis of Fraxetin-responsive genes. Bubble plot showing significantly enriched GO terms. Bubble size represents the number of enriched genes, while color indicates enrichment significance. (**b**) Hierarchical network of Fraxetin’s antifungal mechanisms. The three-layer network illustrates dynamic antifungal processes: Left: Fraxetin-induced damage; Middle: Oxidative stress response with upregulation of antioxidant genes; Right: Membrane-damage pathways with downregulation of phospholipid/sterol synthesis genes. (**c**) Oxidation-centric transcriptomic landscape. Volcano plot highlighting Fraxetin-induced dysregulation of oxidation-related genes.

GO enrichment analysis showed that the differentially expressed genes were primarily concentrated in three core biological processes following Fraxetin treatment (Figure 4a). First, in the Fraxetin damage response, ABC transporters, heat shock proteins, glutathione S-transferases, and various monooxygenases were involved, which are essential for Fraxetin efflux and detoxification. Second, key genes in oxidative stress response, including dehydrogenases, redox enzymes, and peroxidases, participate in maintaining redox balance and electron transfer. Finally, genes related to membrane damage were enriched in membrane transport proteins, phospholipases, and vacuolar sorting proteins, which are involved in maintaining membrane stability and ion balance.

KEGG pathway analysis and the STRING-predicted protein interaction network further confirmed that Fraxetin acted on the pathogenic fungi through three major functional modules (Figure 4b). Fraxetin response module includes transport proteins and heat shock proteins involved in Fraxetin efflux and detoxification; the oxidative stress module involves enzymes regulating redox balance; and the membrane damage response module regulates membrane repair and ion homeostasis. These modules are coordinated through a 5-protein interaction network to collectively regulate the fungal cell’s stress response (Figure 4b, homologues marked with different colored circles, including protein folding quality control: HSP104, HSP82, STI1, AHA1; organelle function and transport: APJ1, JAC1, MGE1, SNL1; metabolic regulation: MGA1; stress response: HSF1, SKN7; and transmembrane transport: SNQ2). The transport protein family (e.g., CFTR/MRP, WHITE, SNQ2) plays a central role in both Fraxetin response and membrane damage modules, mediating Fraxetin efflux and maintaining membrane stability. H^+^-ATPase (F-type) and its subunits (a, c), as well as NADH:quinone reductase, act as a bridge between the oxidative stress and membrane damage modules, regulating proton gradients, energy metabolism, and membrane potential.

Numerous oxidation-related genes were identified through transcriptomic analysis as being upregulated or downregulated in response to Fraxetin treatment (Figure 4c). This finding underscores the crucial role of oxidation-related enzymes in processes like Fraxetin-induced damage, oxidative stress, and cell membrane damage. Fraxetin compromises fungal defenses, causing cellular collapse by disrupting target networks such as efflux, damage repair, and antioxidant systems, pushing fungal cells from adaptation to dysfunction. Oxidation-related enzymes are integral to the maintenance of redox homeostasis, regulation of energy metabolism, and preservation of membrane integrity. Specifically, the interplay between energy and redox processes is mediated by enzymes such as dehydrogenases, NADH: quinone reductases, and H^+^-ATPases. These enzymes are pivotal in facilitating ATP synthesis and establishing proton gradients, both of which are essential for the efflux of Fraxetin and the repair of proteins. In the regulation of redox balance, enzymes such as peroxidases and glutathione reductases operate synergistically to eliminate reactive oxygen species (ROS), thereby mitigating the propagation of oxidative damage. The orchestrated regulation of energy metabolism, ROS elimination, and membrane potential maintenance is crucial for translating Fraxetin-induced damage into quantifiable effects on membrane stability. Collectively, these interconnected processes dictate the overall extent of membrane disruption induced by Fraxetin.

### 3.5. Dose- and Time-Dependent Antifungal Mechanisms of Fraxetin: Oxidative Stress Cascade and Membrane Damage in N. laricinum

**Figure 5:**
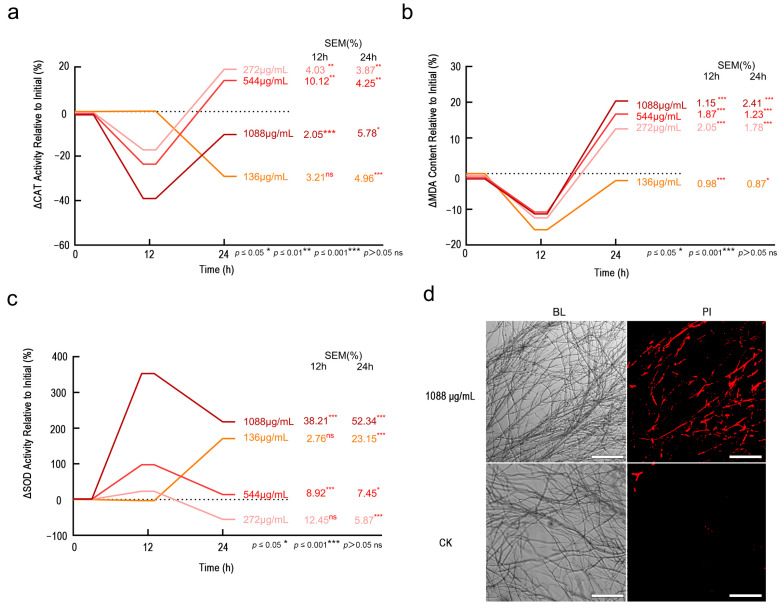
Fraxetin Induces Synergistic Antifungal Activity via Membrane Damage and Biphasic Oxidative Stress in *N. laricinum*. (**a**) Percentage change in CAT activity from pre-Fraxetin treatment levels. (**b**) Percentage change in MDA content from pre-Fraxetin treatment levels. (**c**) Percentage change in SOD activity from pre-Fraxetin treatment levels. (**d**) Propidium iodide (PI) staining of *N. laricinum* hyphae at 24 h post-treatment. Fraxetin (1088 μg/mL) caused extensive red fluorescence, indicating membrane damage and cell death. Control (DMSO) showed intact membranes (no red fluorescence). Scale bars: 200 μm.

Physiological assays confirmed Fraxetin induces fungal death through biphasic lipid peroxidation and oxidative cascade failure. Initially, the impact of varying concentrations of Fraxetin on CAT activity demonstrated a significant inhibition at 12 h, which was followed by a reduction in this inhibitory effect. At 24 h, intermediate concentrations of Fraxetin induced stimulation of CAT activity (272 μg/mL: 18.92%, *p* < 0.001; 544 μg/mL: 13.97%, *p* < 0.001), indicating notable dose- and time-dependent variations (Figure 5a). Administration of a high concentration (1088 μg/mL) of Fraxetin led to a marked inhibition of CAT activity at 12 h (−39.10%, *p* < 0.001), with this suppressive effect persisting at a diminished level after 24 h (−10.30%, *p* < 0.05). Conversely, at a low concentration (136 μg/mL), Fraxetin did not elicit significant changes at 12 h but resulted in a significant suppression at 24 h (−29.10%, *p* < 0.001), suggesting a delayed toxic effect.

Next, various concentrations of Fraxetin demonstrated an inhibitory effect on MDA levels at 12 h, followed by an increase at 24 h. This modulation of MDA levels was dose-dependent (Figure 5b). At a low concentration (136 μg/mL), Fraxetin maintained a persistent suppression of MDA levels at 12 h, although this inhibition diminished by 24 h, resulting in a rebound of MDA levels. Medium concentrations (272 μg/mL and 544 μg/mL) also reduced MDA levels at 12 h but led to an activation of MDA levels at 24 h. Notably, a high concentration (1088 μg/mL) of Fraxetin resulted in a significant inhibition of MDA levels at 12 h (−11.28%, *p* < 0.001), yet significantly activated MDA levels at 24 h (+20.35%, *p* < 0.001). This suggests that lipid peroxidation reached its peak at 24 h, potentially due to the accumulation of ROS.

In terms of SOD activity, exposure to a high concentration of Fraxetin (1088 μg/mL) elicited a pronounced activation effect, evidenced by a substantial increase in SOD activity at 12 h (+353.10%, *p* < 0.001), which remained elevated at 24 h (+217.70%, *p* < 0.001) (Figure 5c). This observation indicates that the fungus may respond to acute oxidative stress by markedly enhancing SOD activity to mitigate superoxide radicals. At medium concentrations (272 μg/mL and 544 μg/mL), Fraxetin induced a moderate activation of SOD activity at 12 h, followed by inhibition at 24 h. Conversely, at a low concentration (136 μg/mL), Fraxetin significantly enhanced SOD activity at 24 h (+170.80%, *p* < 0.001).

PI staining revealed that the treatment group receiving 1088 μg/mL exhibited the highest proportion of red-stained hyphae, indicative of substantial membrane damage and cell death.

This observation suggests that elevated concentrations of Fraxetin compromise the cell membranes of pathogenic fungi, whereas the control group demonstrated negligible staining (Figure 5d). These physiological changes align with the gene expression alterations identified in the transcriptomic analysis, which were enriched in pathways such as glycerophospholipid metabolism (ko00564) and sterol biosynthesis (ko00100). Furthermore, these physiological changes are corroborated by physiological assays measuring CAT, MDA, and SOD activity. The study indicates that the impact of Fraxetin on *N. laricinum* is dependent on both dose and time. At high doses, Fraxetin inhibits CAT and activates SOD, resulting in oxidative stress and membrane damage. Medium doses activate antioxidant defenses through SOD and CAT. Conversely, low doses delay toxicity but ultimately result in chronic toxicity.

## 4. Discussion

The primary aim of this study was to assess the antifungal efficacy of the disease-induced phytoalexin Fraxetin against *N. laricinum* and to investigate the hypothesis that its mechanism of action involves the disruption of fungal membrane integrity through lipid peroxidation. Our results substantiate this hypothesis, revealing that Fraxetin’s effectiveness is attributable to its capacity to initiate an oxidative cascade within the pathogen. This cascade is triggered by a disruption of redox homeostasis, leading to extensive lipid peroxidation, which ultimately causes irreversible membrane damage and cell death. This research not only elucidates a complex chemical defense mechanism in larch but also provides a scientific basis for the development of Fraxetin as a sustainable alternative to conventional fungicides, presenting a promising strategy for the environmentally friendly management of larch shoot blight.

The catechol core of Fraxetin imparts significant reducing activity [30], and its tendency to lose electrons and form quinone structures is well-documented [19]. As a reductant, Fraxetin is capable of scavenging reactive oxygen and nitrogen species (ROS/RNS) and terminating radical chain reactions, thereby safeguarding biomolecules [31,32,33]. Nonetheless, previous studies have highlighted that cellular viability is contingent upon a precisely balanced redox state, with excessive reductive stress being inherently cytotoxic [34,35]. This cytotoxicity arises partly from the disruption of redox signaling and the attenuation of antioxidant defenses such as SOD, glutathione peroxidase (GPx), and glutathione [36,37]. Consistent with existing literature, our transcriptomic analysis suggests that Fraxetin disrupts fungal redox homeostasis, leading to oxidative stress. This phenomenon can be attributed to well-established chemical principles: reductants that facilitate the reduction of metal ions (e.g., Fe^3+^ to Fe^2+^) enhance Fenton reactions with H_2_O_2_, resulting in the production of hydroxyl radicals [38]. These radicals initiate lipid peroxidation, compromise membrane integrity, and generate deleterious aldehydes and hydroperoxides [39]. In agreement with previous studies on catechols, the catechol moiety of Fraxetin is capable of inducing significant ROS bursts and lipid peroxidation through metal redox cycling [40]. Additionally, the hydrophobic nature of the coumarin scaffold promotes membrane insertion [41,42], while its phenolic hydroxyl groups can disrupt proton gradients and membrane potential [43]. Similar to earlier findings, fungi employ GSH, SOD, and CAT as defensive mechanisms against ROS; however, depletion of these defenses exacerbates oxidative damage and membrane disruption. Consequently, our observation that Fraxetin, a chemically reducing agent, results in fungal oxidation highlights a context-dependent transition from radical scavenging to redox cycling-induced ROS production. This aligns with previous evidence and elucidates the fungicidal effects observed.

Mechanistically, the observation that a chemically reducing Fraxetin results in fungal oxidation can be attributed to its catechol-driven redox cycling. The catechol structure plays a crucial role in inducing lethal oxidative stress, characterized by a burst of ROS and lipid peroxidation. This occurs as catechols reduce metal ions, thereby initiating the Fenton reaction to produce ·OH that directly attack membrane lipids [40]. This mechanism aligns with previous studies on catechols and is further supported in our system by two membrane-centric characteristics previously identified in coumarins: the hydrophobic coumarin scaffold integrates into the lipid bilayer, causing physical disruption of membrane architecture [41,42], while phenolic hydroxyl groups function as proton carriers and hydrogen bond donors, disrupting membrane potential and proton gradients, thus increasing permeability [43]. Consistent with previous research, fungal cells initially mitigate the ROS induced by Fraxetin through the mobilization of antioxidants, such as glutathione (GSH), and antioxidant enzymes. The distinct kinetics of SOD and CAT reflect different phases of this response. A marked activation of SOD indicates an immediate defense against the initial surge of superoxide radicals [44]. In contrast, CAT experiences suppression, transient compensatory imbalance, and eventual failure, suggesting an inability to neutralize secondary ROS, specifically H_2_O_2_. This failure is attributed to direct inhibition, saturation or inactivation at elevated H_2_O_2_ levels, and depletion of resources [45]. Upon depletion of the antioxidant system, ROS accumulation intensifies, resulting in irreversible membrane lipid damage and subsequent cell lysis under osmotic stress. Building upon previous models, our transcriptomic analysis identifies membrane damage as the primary event, evidenced by the downregulation of key lipid metabolism genes such as ERG3 and CDS/PSS. This downregulation leads to an imbalance in sterol/phospholipid composition, increased positive PI staining, and reduced secretion of virulence factors. Consequently, a causal pathway is established from redox cycling-induced ROS to membrane failure and diminished lesion development.

Prior to its initiation, we evaluated the toxicity of Fraxetin to larches using a concentration of 4000 μg/mL and confirmed that it exerts no toxicity on the host (Table S1). On this safe basis, this study confirms that Fraxetin’s antifungal activity is attributable to redox cycling-induced lipid peroxidation and subsequent membrane disruption. However, three limitations constrain the interpretation of these findings and suggest directions for future research. Firstly, the transport and fate of Fraxetin within plant systems require further elucidation. Although coumarins are known to penetrate and diffuse through plant tissues via dual transport mechanisms and achieve long-distance movement through the xylem and phloem—accounting for the efficacy of exogenous Fraxetin within larch [46]—the spatiotemporal dynamics, chemical speciation (e.g., conjugation), and local concentrations at infection sites, as well as the specific interaction sites with fungal membrane proteins (e.g., ABC transporters) within the plant, remain inadequately defined. Secondly, the ecological context was not assessed; plant–microbe interactions in natural settings may influence efficacy. This includes the role of beneficial rhizosphere microbes, such as mycorrhizae, in modulating Fraxetin accumulation, the potential antagonistic or synergistic interactions among pathogenic communities and non-target soil microbes that may alter antifungal activity, and the impact of dynamic changes in microbial community structure on stability and bioavailability. Addressing these gaps would benefit from elucidating Fraxetin–membrane protein complexes to guide molecular optimization and from developing nanocarrier delivery systems to enhance stability and xylem targeting. Thirdly, practical implementation is hindered by economic constraints: the relatively high cost of high-purity Fraxetin limits its immediate application in field settings. This necessitates the exploration of crude Fraxinus spp. extracts as more viable spray formulations, validation across diverse larch genotypes and pathosystems, and comprehensive techno-economic and life cycle analyses. These limitations collectively delineate the subsequent phase of this research, which involves the quantitative mapping of Fraxetin transport, transformation, and target engagement within plants. This phase also includes microbiome-aware field trials to elucidate the ecological modulation of efficacy, alongside cost-sensitive formulation strategies. These efforts aim to enhance the mechanistic understanding and expedite the translation of findings into sustainable management practices for larch shoot blight.

For hemibiotrophic pathogens, phytoalexins are key in early local defense responses (LDR): they accumulate rapidly at infection sites, acting as the first line of defense by inhibiting early pathogen spread and local disease progression [47,48]. Meanwhile, plant hormones regulate systemic acquired resistance (SAR), which—unlike phytoalexins’ immediate local role—focuses on long-term defense, aiding faster, more effective responses to secondary infections and lowering reinfection risk [49,50]. Thus, phytoalexins’ immediate LDR inhibition and hormone-regulated SAR’s secondary infection prevention, with distinct focuses but equal importance, together form a synergistic defense system against these pathogens [51]. In summary, Fraxetin exerts antifungal actions that provide mechanistic insights into the study of botanical fungicides. It significantly inhibits *N. laricinum* infection through a mechanism driven by membrane damage, coupled with dynamic regulation of oxidative stress. In addition, Fraxetin displays unique resistance properties by modulating antioxidant enzyme activity and directly inhibiting pathogen growth. The synergistic effects of these actions offer new molecular targets for the development of botanical fungicides and biomarkers for resistant breeding.

## Figures and Tables

**Figure A1:**
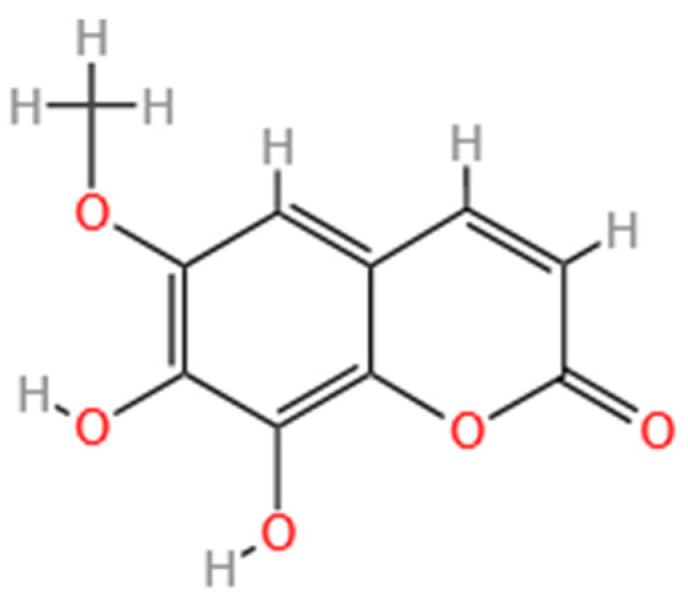
The 2D structure of Fraxetin.

## Data Availability

The original contributions presented in this study are included in the article/Supplementary Material. Further inquiries can be directed to the corresponding authors.

## Supplementary Materials

The following supporting information can be downloaded at: https://www.mdpi.com/article/10.3390/jof11100724/s1, Table S1: Host Toxicity Test of Fraxetin on Larch.
