# Supplementary material for: The Mechanism of Fraxetin as a Sustainable Fungicide for Larch Shoot Blight: Lipid Peroxidation and Oxidative Stress in Neofusicoccum laricinum
Source: J Fungi (Basel). 2025 Oct 8;11(10):724. doi: 10.3390/jof11100724 (PMC12565604; doi:10.3390/jof11100724)
Supplement: Supplementary file 1 [file jof-11-00724-s001.zip › jof-3878652-supplementary.pdf]

Table S1. Host Toxicity Test of Fraxetin on Larch.

Treatment	Health Score
Fraxetin	80
Fraxetin	80
Fraxetin	90
CK	100
CK	100
CK	80
